# A new golden frog species of the genus *Diasporus* (Amphibia, Eleutherodactylidae) from the Cordillera Central, western Panama

**DOI:** 10.3897/zookeys.196.2774

**Published:** 2012-05-21

**Authors:** Andreas Hertz, Frank Hauenschild, Sebastian Lotzkat, Gunther Köhler

**Affiliations:** 1 Senckenberg Forschungsinstitut und Naturmuseum, Senckenberganlage 25, 60325 Frankfurt am Main, Germany; 2 Johann Wolfgang Goethe-University, Institute for Ecology, Evolution and Diversity, Biologicum, Building C, Max-von-Laue-Straße 13, 60438 Frankfurt am Main, Germany

**Keywords:** Central America, Anura, diversity of species, taxonomy, vocalization

## Abstract

We describe the frog species *Diasporus citrinobapheus* **sp. n.** from the Cordillera Central of western Panama. The new species differs from all other species in its genus in coloration, disk cover and disk pad shape, skin texture, advertisement call, and size. It is most similar to *Diasporus tigrillo*, from which it differs in dorsal skin texture, relative tibia length, number of vomerine teeth, ventral coloration, dorsal markings, and relative tympanum size, and to *Diasporus gularis*, from which it can be distinguished by the lack of membranes between the toes, adult size, posterior thigh coloration, and position of the choanae. We provide data on morpho- logy, vocalization, and distribution of the new species, as well as brief information on its natural history.

## Abstract (es)

Describimos la especie de rana *Diasporus citrinobapheus* **sp. n.** de la Cordillera Central, occidente de Panamá. La nueva especie se distingue de otras especies del género por su coloración, su forma de la cubierta y la almohadilla de los discos, textura de la piel, canto de anúncio, y tamaño corporal. Se asemeja mas a *Diasporus tigrillo*, del cual se distingue por la textura de la piel dorsal, longitud relativa de la tibia, número de dientes vomerianos, coloración ventral, patrón dorsal, y tamaño relativo del tímpano, y a *Diasporus gularis*, del cual se diferencia por la ausencia de membranas entre los dedos de pie, tamaño corporal, coloración de la parte trasera del muslo, y posición de las coanas. Presentamos datos de la morfología, vocalización, y distribución de la nueva especie, así como notas concisas de su historia natural.

## Introduction

Panama’s herpetofauna is known to be the most diverse in consideration of its size in Central America, with only Mexico being more diverse in absolute species count (Myers and Duellman 1982; Jaramillo et al. 2010). Although herpetological research has been conducted in Panama for more than a hundred years (Ibáñez et al. 2001), the knowledge of amphibian species diversity is still far from being completed. This is demonstrated impressively by the multitude of amphibian species described from this country within the last years (e.g. Wake et al. 2005, 2007; Köhler et al. 2007; Mendelson et al. 2008; Bolaños and Wake 2009; Crawford et al. 2010b; Mendelson and Mulcahy 2010; Ryan et al. 2010a, 2010b).

The genus *Diasporus* (Hedges et al. 2008) is the closest relative of the predominantly Caribbean genus *Eleutherodactylus*. The species of *Diasporus* are distributed from eastern Honduras to western Ecuador (Frost 2011; Köhler 2011). The genus contains nine described species, five of which (*Diasporus diastema* Cope, *Diasporus hylaeformis* Cope, *Diasporus tigrillo* Savage, *Diasporus ventrimaculatus* Chaves, García-Rodríguez, Mora and Leal, and *Diasporus vocator* Taylor) are currently known to occur in western Panama and/or southern Costa Rica. The remaining four species (*Diasporus anthrax* Lynch, *Diasporus gularis* Boulenger, *Diasporus quidditus* Lynch, and *Diasporus tinker* Lynch) are distributed in Panama east of the Canal, and further along the Pacific coast of northern South America southwards to northwestern Ecuador (Frost 2011; IUCN 2011). However, differences in body size, male advertisement call, and coloration in the genus *Diasporus* suggest that there are several undescribed species (Lynch and Duellman 1997; Ibáñez et al. 1999; Savage 2002). Recent fieldwork in the Serranía de Tabasará of western Panama resulted in the discovery of a remarkable new species of *Diasporus*. The purpose of this paper is to describe this new species.

## Materials and methods

Field work was carried out between May and August 2010 at several sites along both slopes of the Serranía de Tabasará between the Fortuna depression and Santa Fé, Veraguas, Panama. All specimens were encountered during opportunistic searches at night. Preparation and preservation of voucher specimens follows Köhler (2001). Tissue samples, usually the left forearm, were stored in 98% undenatured ethanol and deposited in the tissue collection of the Senckenberg Forschungsinstitut und Naturmuseum Frankfurt, Germany (SMF). Geographic coordinates and altitude above sea level were recorded with a handheld Garmin GPS MAP 60 CSx GPS receiver. All georeferences are in the geographical coordinate system and WGS 1984 datum, given in decimal degrees rounded to the fourth decimal place. Elevations are rounded to the next tenth. The map was created using ArcGIS 10 (ESRI).

We took additional morphological data from all Central American species currently assigned to the genus *Diasporus* in the SMF collection. We list all specimens examined for comparison in the Appendix I. Abbreviations for museum collections follow those of Sabaj Pérez (2010) except MHCH (Museo Herpetológico de Chiriquí, the herpetological collection of the Universidad Autónoma de Chiriquí, David, Panama). Specimens in the Appendix labeled with AH field numbers will be deposited at MHCH.

The sex of the male holotype and the paratypes was determined by the presence of vocal slits and vocal sac. Measurements were made with a dial caliper with the aid of a dissecting microscope and rounded to the nearest 0.1 mm. Measurements of the holotype (LACM 146212) and paratype (LACM 146241) of *Diasporus tigrillo* were taken from Savage (1997), those of *Diasporus ventrimaculatus* from Chaves et al. (2009). Additionally, we examined photographs of the type specimens of *Diasporus tigrillo*. If possible, missing measurements were calculated on the basis of data presented in the respective descriptions. In the case of *Diasporus ventrimaculatus*, no individual measurements are available, for which reason calculations were made using the average values given for the paratypes by Chaves et al. (2009). We follow Savage (1997, 2002) in the terminology of disk cover and disk pad shape, dorsal outline of head, and snout profile shape. Abbreviations used for measurements are: snout-vent length (direct line distance from the tip of the snout to the posterior margin of the vent): SVL; length of Finger III (from distal end of the Finger III including disk to the base of the second subarticular tubercle): LF III; length of Toe IV (from distal end of the toe IV including disk to the base of third subarticular tubercle): LT IV; disk width at Finger III (at greatest width): DWF III; disk width at Toe IV (at greatest width): DWT IV; head length (from quadratojugal region to tip of the snout): HL; head width (between angles of jaw): HW; tibia length (straight length of the tibia): TL; horizontal eyelid diameter (greatest length of the upper eyelid): EL; interorbital distance (the width of frontoparietal bone between the orbits): IOD; horizontal tympanum diameter (at greatest length): TD; and horizontal eye diameter (at greatest length): ED. The capitalized colors and color codes (the latter in parentheses) in color descriptions in life are those of Smithe (1975–1981). Drawings of head, hands, and feet were made using a camera lucida attachment for a Leica MZ 12 dissecting microscope. Values provided for morphometric and acoustic para- meters are minimum, maximum, and mean value ± standard deviation.

Advertisement calls were recorded using a Sennheiser ME 66 shotgun microphone capsule with a Sennheiser K6 powering module in combination with the Marantz PMD 620 solid-state recorder. A minimum distance from microphone to frog of one meter was kept while recording to prevent disturbance. As needed, the microphone was attached to branches with the aid of a Joby Gorillapod in order to minimize disturbance of the calling frog. Calls were recorded in PCM format at a sampling rate of 48 kHz with 24 bit resolution and stored as wav files on a SD Card. Call editing and analysis were performed using SOUND RULER 0.9.6.0 (Gridi-Papp 2003–2007) for frequency analysis and to generate figures of oscillograms and audiospectrograms. We measured temporal parameters by hand using ADOBE AUDITION 3.0, because SOUND RULER has difficulties in accurately and precisely measuring temporal parameters (Bee 2004). Frequency information was obtained through Fast Fourier Transformation (FFT length 512 points, overlap between FFTs 0.8) at Hanning window function. Air temperature and humidity were measured immediately after each sound recording using the digital device Voltcraft HT-200 to the nearest 1°C and 3.5% relative humidity (RH). An alcohol extraction of skin secretions of the new species has been examined for alkaloids using Liquid Chromatography-Time-Of-Flight-Mass Spectrometry (LC-TOF-MS) at the Center of Forensic Medicine of the Goethe-University Frankfurt am Main.

For the complementary molecular analysis, we extracted DNA following the protocol of Ivanova et al. (2006). To eliminate potential PCR-inhibiting contaminants, the tissue samples were incubated for one hour in TE-buffer (10 parts Tris-HCl (pH = 8.0) and one part EDTA) before digestion for at least 10 hours in 50 µl Vertebrate Lysis Buffer and 5.2µl Proteinase K at 56 °C. After extraction, DNA was eluted in 50 µL TE buffer. A fragment of the mitochondrial 16S rRNA gene was amplified in an MJ Research Dyad Deciple™ Peltier Thermal Cycler using the following program: initial denaturation for 180 s at 94 °C; followed by 39 cycles with denaturation for 15 s at 94 °C, hybridization for 60 s at 51 °C, and elongation for 60 s at 72 °C; final elongation for 120 s at 72 °C. Reaction mix for each sample contained 1 µL DNA template, 2.5 µL Reaction Buffer (PeqGold), 2.5 µL 2.5 mM dNTPs, 0.5 µL Taq Polymerase (PeqLab), 16.5 µL H_2_O, and 1 µL of each primer (forward: 16SA-L, 5’-CGCCTGTTTATCAAAAACAT-3’; reverse:16SB-H, 5’-CCGGTCTGAACTCAGATCACGT-3’). The achieved 16S sequences were deposited in GenBank. We compared 21 sequences of the genus *Diasporus* in our analysis, three of the type series of the new species and one referred specimen, four specimens referred to as *Diasporus aff. hylaeformis* from Cerro Pando, as well as all16S sequences of the genus *Diasporus* available on GenBank. We used an additional 16S sequence of *Pristimantis ridens* as an outgroup (see Appendix II for examined specimens and GenBank accession numbers). Sequences were aligned with ClustalW (Larkin et al. 2007) using the default settings in Geneious (Drummond et al. 2010). The manually refined final alignment contained 535 positions. Using MEGA5 (Tamura et al. 2011), we computed uncorrected pairwise genetic distances, determined the Tamura 3-parameter model (Tamura 1992) as the best-fitting substitution model, and conducted Maximum Likelihood as well as Maximum Parsimony analyses (each with 10000 bootstrap replicates). Using TCSv1.21 (Clement et al. 2000), we conducted a statistical parsimony network analysis, with gaps considered as a fifth character state and a connection limit of 95%.

## Results

### Diasporus citrinobapheus sp. n.

urn:lsid:zoobank.org:act:4A526693-CA45-44FC-9D9D-4F3064A47341

http://species-id.net/wiki/Diasporus_citrinobapheus

**Figure 1.**
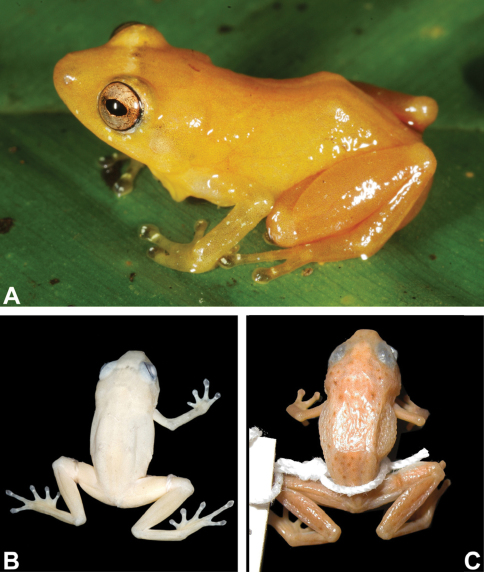
**A–B** Holotype of*Diasporus citrinobapheus* (SMF 89814, adult male): **A**in life **B** in preservative. **C** *Diasporus tigrillo* in preservative (LACM 146212, holotype, adult male), note dark brown spots. Pictures are not at the same scale.

Figures 1 A, B 2 3 5

#### Holotype.

Adult male SMF 89814: collected on June 26, 2010 at 19:13 by Andreas Hertz and Sebastian Lotzkat at Quebrada Rasca (8.4851°N, 81.1727°W, 790 m elevation), near Paredón, Comarca Ngöbe-Buglé, western Panama, approximately 50 airline km NNW of the city of Santiago and 20 airline km N of Cañazas, Veraguas.

#### Paratypes.

All collected by Andreas Hertz and Sebastian Lotzkat at the type locality on June 26, 2010: MHCH 2370-71; SMF 89816; all adult males.

#### Referred specimens.

Adult males SMF 89817 and MHCH 2372: collected on July 01, 2010 by Andreas Hertz and Sebastian Lotzkat at the private reserve Willie Mazú, Comarca Ngöbe-Buglé (8.7903°N, 82.1989°W, 681 m elevation); female SMF 89820: collected on March 31, 2009 by Andreas Hertz, Sebastian Lotzkat and Arcadio Carrizo at Cerro Negro, Parque Nacional Santa Fé, Veraguas (8.5691°N, 81.0988°W, 730 m elevation).

#### Diagnosis.

A member of the genus *Diasporus* based on the following combination of characters: vocal slits and a single subgular vocal sac present, adult males without nuptial thumb pads; Finger I shorter than Finger II; Toe III much shorter than Toe V; subarticular tubercles on hands and feet flattened; no supernumerary tubercles on hands and feet; no tarsal fold or tubercle. *Diasporus citrinobapheus* differs from alldescribed members of its genus by the following combination of characters (for accounts, see Table 1): coloration bright yellow to orange in life (Fig. 1 A); head almost as broad as long, but comparatively broad in relation to SVL; skin of dorsum smooth; venter coarsely areolate; tympanum covered by skin but annulus clearly visible; TD about 41% of ED; EL on average narrower than IOD; snout subacuminate in profile and rounded to subovoid in dorsal outline; disks of fingers and toes slightly expanded, disk covers of most fingers and toes spadate, but lacking papillae; disk pads of most fingers and toes triangular; subarticular tubercles of hands and feet rounded, very flat, almost not visible; vomerine odonthophores longish oval and widely separated; vomerine teeth weakly developed; upper eyelid usually smooth, very low pustules in some individuals; heel smooth. Its bright yellow to orange coloration distinguishes *Diasporus citrinobapheus* from almost all described Central American *Diasporus*, which, in spite of considerable variation, are all tan to gray or brownish to almost black. In *Diasporus hylaeformis* and *Diasporus ventrimaculatus*, the dorsal ground color can be suffused with pink or red. Only *Diasporus tigrillo* from Costa Rica, a species known only from two specimens, shows a yellowish coloration in life according to the original description (Savage 1997). *Diasporus citrinobapheus* differs from the two known specimens of *Diasporus tigrillo* in the following characters (character for *Diasporus tigrillo* in parentheses): SVLinadult males 17.3–19.7 mm (16.0–17.5 mm); dorsal skin absolutely smooth (dorsal skin with scattered low pustules, best developed on dorsum); TD 32–45% of ED (54–57%); TL 40% of SVL (about 48%); distal subarticular tubercle of Finger and Toe I flat and rounded (weakly bifid); many weakly developed vomerine teeth in three to four close rows (a few vomerine teeth in two obliquely aligned and widely separated rows); dorsal surface uniformly bright yellow to orange, sometimes with irregularly distributed dark blotches (yellow to orange dorsal coloration with dark brown spots confined to the pustules); ventral surfaces almost colorless and transparent, in some individuals with a fine dirty white speckling, except male vocal sac that is suffused with yellow (undersurfaces, including venter, yellow); coloration in preservative grayish-white with only a suggestion of yellow (brownish-ocher with dark brown dots; see comments in Discussion section for the usage of different preservation methods). Furthermore, *Diasporus citrinobapheus* superficially resembles the South American *Diasporus gularis* from western Ecuador and western Colombia in coloration (see photo in Lynch 2001, page 295 Fig. 7). *Diasporus gularis* has been described comprehensively by Lynch and Duellman (1997). According to them, adult *Diasporus gularis* are larger (SVL in males 20.2–21.6 mm, in females 23.3–24.8 mm) than *Diasporus citrinobapheus* (males 17.3–19.7 mm, single known female 21.8 mm). Moreover, *Diasporus gularis* shows basal webbings between toes and some specimens have papillae at the apex of the disk pad on some toes, whereas there are no such papillae, and no webbing between toes of *Diasporus citrinobapheus*. The posterior surfaces of thighs are brown in *Diasporus gularis*, but yellow to orange in *Diasporus citrinobapheus*. Moreover, the choanae are long, oval, and not concealed by the palatal shelf of the maxillary arch in *Diasporus gularis*, whereas they are round, orientated extremely laterally on palate, and partially concealed by the palatal shelf of the maxillary arch in *Diasporus citrinobapheus*.

**Table 1.** Morphological measurements of *Diasporus citrinobapheus* in comparison with other described species of the genus from western Panama and southern Costa Rica (mean±SD, min–max). See Materials and Methods for abbreviations.

**Character**	***Diasporus citrinobapheus***		***Diasporus diastema***		***Diasporus hylaeformis***		***Diasporus ventrimaculatus***		***Diasporus vocator***		***Diasporus tigrillo***
	male (n=6)	female (n=1)	male (n=20)	female (n=22)	male (n=9)	female (n=5)	male (n=6)	female (n=2)	male (n=4)	female (n=6)	male (n=2)
SVL (mm)	18.7±0.63 (17.3–19.7)	21.8	18.7±1.62 (15.9–22.1)	18.7±2.58 (15.0–23.5)	19.1±1.30 (16.9–20.9)	21.2±0.97 (19.2–21.7)	21.8±1.2 (20.2–23.5)	23.9±0.8 (23.2–24.7)	15.3±2.18 (12.2–17.2)	14.7±2.18 (13.6–15.7)	16.8 (16.0–17.5)
DW/LF III	0.23±0.03 (0.18–0.26)	0.23	0.36±0.06 (0.21–0.44)	0.32±0.07 (0.21–0.44)	0.31±0.03 (0.27–0.36)	0.32±0.02 (0.29–0.34)	-	-	0.26±0.06 (0.19–0.32)	0.32±0.06 (0.24–0.42)	-
DW/LT IV	0.14±0.03 (0.11–0.18)	0.17	0.23±0.05 (0.15–0.32)	0.22±0.05 (0.11–0.29)	0.22±0.03 (0.18–0.26)	0.22±0.02 (0.20–0.24)	-	-	0.17±0.03 (0.13–0.19)	0.17±0.03 (0.14–0.19)	-
HL/SVL	0.41±0.01 (0.39–0.44)	0.40	0.39±0.02 (0.35–0.44)	0.41±0.02 (0.36–0.44)	0.39±0.02 (0.35–0.43)	0.39±0.02 (0.37–0.42)	0.33	0.35	0.38±0.02 (0.35–0.41)	0.38±0.02 (0.35–0.42)	0.39 (0.38–0.40)
HW/SVL	0.37±0.01 (0.35–0.38)	0.36	0.36±0.02 (0.33–0.39)	0.36±0.02 (0.32–0.39)	0.37±0.01 (0.35–0.39)	0.36±0.02 (0.35–0.40)	0.39	0.40	0.34±0.02 (0.31–0.36)	0.34±0.02 (0.31–0.36)	0.36 (0.34–0.37)
HW/HL	0.91±0.03 (0.88–0.97)	0.90	0.91±0.06 (0.79–1.01)	0.90±0.06 (0.78–1.04)	0.94±0.05 (0.85–1.00)	0.92±0.04 (0.85–0.96)	1.15	1.14	0.89±0.08 (0.79–0.96)	0.89±0.08 (0.86–0.95)	0.92 (0.85–0.99)
TL/SVL	0.41±0.01 (0.40–0.42)	0.42	0.40±0.04 (0.35–0.51)	0.42±0.05 (0.36–0.56)	0.39±0.01 (0.37–0.42)	0.39±0.05 (0.35–0.47)	0.50	0.51	0.40±0.02 (0.38–0.43)	0.38±0.02 (0.36–0.42)	0.48 (0.46–0.50)
EL/IOD	0.98±0.12 (0.83–1.12)	0.94	1.04±0.10 (0.89–1.24)	1.12±0.18 (0.89–1.62)	1.01±0.12 (0.88–1.24)	1.07±0.10 (0.88–1.19)	0.86	1.00	1.07±0.12 (0.95–1.24)	1.43±0.12 (1.25–1.59)	-
ED/HL	0.32±0.03 (0.28–0.36)	0.32	0.29±0.04 (0.22–0.35)	0.29±0.04 (0.21–0.37)	0.30±0.03 (0.27–0.35)	0.28±0.03 (0.22–0.30)	0.37	0.39	0.33±0.01 (0.32–035)	0.34±0.01 (0.33–0.37)	0.32 (0.28–0.35)
TD/ED	0.39±0.07 (0.32–0.45)	0.32	0.38±0.09 (0.27–0.65)	0.37±0.08 (0.19–0.52)	0.42±0.07 (0.30–0.52)	0.45±0.03 (0.42–0.50)	0.48	0.47	0.36±0.07 (0.30–0.44)	0.43±0.07 (0.33–0.50)	0.55 (0.54–0.57)
ED/SVL	0.13±0.01 (0.11–0.15)	0.13	0.11±0.20 (0.08–0.13)	0.12±0.13 (0.09–0.15)	0.12±0.01 (0.10–0.14)	0.11±0.01 (0.09–0.12)	0.12	0.13	0.13±0.01 (0.12–0.13)	0.13±0.01 (0.12–0.14)	0.12 (0.11–0.13)

#### Description of the holotype.

**Figure 2.**
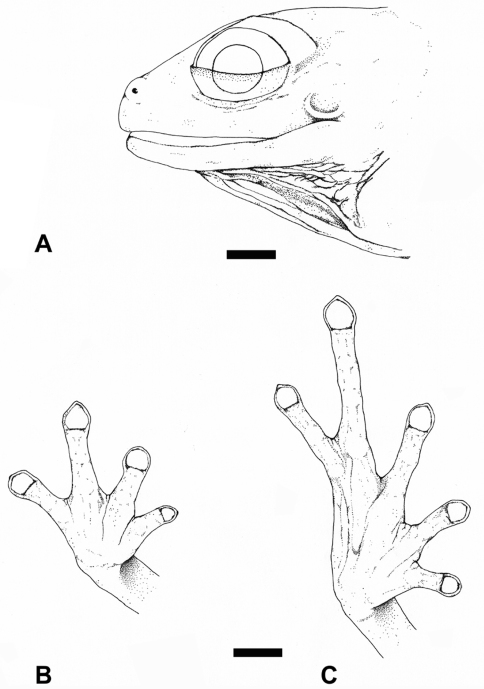
Holotype of*Diasporus citrinobapheus* (SMF 89814, adult male): **A** Lateral view of head **B** Ventral view of right hand. **C** Ventral view of right foot. Scale bars = 1 mm.

An adult male; measurements (in mm): SVL 18.4, LF III 2.4, LT IV 4.2, DWF III 0.6, DWT IV 0.5, HL 7.2, HW 7.0, TL 7.8, EL 2.6, IOD 2.9, TD 0.8, ED 2.4; dorsal skin smooth; venter coarsely areolate; no discoidal fold; upper eyelid smooth; snout subovoid in dorsal outline and subacuminate in profile; nostrils weakly protuberant, directed dorsolaterally; head slightly longer than wide, width 97% of length; HW 38% of SVL; canthus rostralis indistinct; ED 36% of HL and 13% of SVL; EL 90% of IOD; TD 33% of ED (Fig. 2 A); choanae round, orientated extremely laterally on palate, partially concealed by palatal shelf of maxillary arch; elliptical vomerine odonthophores, posteromedian to choanae, which are widely separated from each other, with four rows of weakly developed, short teeth; legs short in relation to body; TL 42% of SVL; relative finger length: I<II=IV<III; all fingers with disks, slightly wider than digits, on Fingers II–IV wider than on Finger I; relative toe length: I<II<III<V<IV, Toe V much longer than toe III; tip of Toe V extending to distal subarticular tubercle on Toe IV; tip of Toe III extending to penultimate subarticular tubercle on Toe IV; disks on Toes III–V larger than on I–II; disk covers spadate, lacking papillae; no supernumerary tubercles (Figs 2 B,C).

#### Etymology.

The specific name *citrinobapheus* is a noun in apposition and is derived from the Greek words *citrinos* (citrin-yellow) and *bapheus* (dyer) referring to the yellow body color that dyes one’s fingers yellowish when the frog is handled. Although we could observe this phenomenon in a few other species of *Diasporus* too, it is notably evident in the new species.

#### Coloration in life.

**Figure 3.**
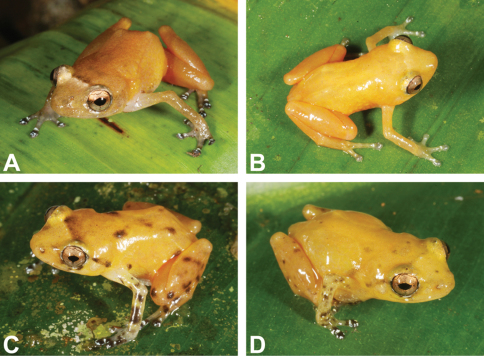
Variation in coloration pattern in life of *Diasporus citrinobapheus* from different localities: **A** Female SMF 89820 from Cerro Negro, Parque Nacional de Santa Fé (Veraguas, Panama) with dirty orange coloration **B** Male SMF 89816 from type locality Paredón (Comarca Ngöbe-Buglé, Panama) with immaculate yellow coloration **C** Male MHCH 2372 from Willie Mazú (Comarca Ngöbe-Buglé, Panama) with intense mottling **D** Male SMF 89817 from Willie Mazú (Comarca Ngöbe-Buglé, Panama) with intermediate mottling.

All examined specimens show shades of bright yellow and orange dorsally; some have dark grayish and/or whitish-grayish spots (Fig. 3). Ventral surfaces are almost achlorophyllaceous and transparent apart from the yellow male vocal sac.

MHCH 2372 (Fig. 3 C): Dorsal ground color Orange Yellow (18); posterior and anterior surfaces of thighs Chrome Orange (16); Raw Umber (23) interorbital and postocular stripes formed by very fine mottling; dorsum with five Dark Grayish Brown (20) blotches, forming a pattern like the five dots on a dice; scattered Dark Grayish Brown (20) blotches on dorsal surfaces of limbs; disk covers Blackish Neutral Gray (82), with white rings at the base; ventral surface of hind limbs Chrome Orange (16); ventral surface of body transparent with dirty white mottling; vocal sac white with a suggestion of Spectrum Yellow (55).

SMF 89820 (Fig. 3 A): In the only female, coloration in life has been recorded as follows: Dorsal surface Yellow Ocher (123 C); a Chamois (123 D) interorbital bar; anterior and posterior surfaces of thighs Chrome Orange (16); venter almost transparent; upper surfaces of disks Sepia (119) with dirty white spots and a dirty white ring around base; gular region Smoke Gray (44).

#### Coloration in preservative (70% alcohol).

In preservation the bright yellow and orange colors fade rapidly to a pale grayish yellow (Fig. 1 B) with scattered dark grayish blotches in some individuals. Legs pale orange; vocal sac pale yellow in males; gular area in females pale gray; tips of digits dark grayish black. Dark grayish black eyeballs shining through skin when head is viewed dorsally.

#### Variation.

Compared to other species of this genus, the individuals of *Diasporus citrinobapheus* available to us exhibit only little variation in their coloration (Fig. 3). All show a yellow to orange dorsal ground color in life. This can either appear bright and clear or somewhat dirty, depending on the pigment translocation within the melanophores in the frog’s skin. In some individuals, higher concentrations of melanophores in certain areas of the dorsum form dark blotches or stripes. This is especially the case in the two specimens from Willie Mazú (Figs 3 C, D). The most frequent pattern of this type is an interorbital bar, which in most cases is darker than ground color along the anterior edge of the bar and lighter than ground color along the posterior edge. In addition, some individuals show dark brown blotches on the limbs and less frequently also on the dorsum. Most individuals show additional small whitish spots, in particular under and around the eyes, as well as scattered across the forelimbs. In the male SMF 89816 from the type locality (Fig. 3 B) the dark and white markings on and around the disk covers are not as pronouncedly contrasting as in the other individuals examined.

#### Molecular genetics.

**Figure 4.**
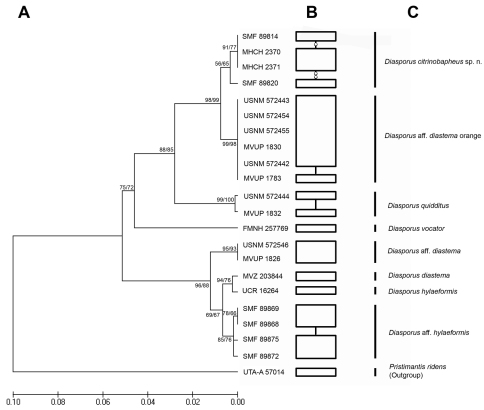
Results of 16S mtDNA analysis. **A** Consensus tree from Maximum Likelihood analysis. Scale bar refers to substitutions per site. Bootstrap support values before the slash correspond to Maximum Likelihood analysis, those after the slash to the Maximum Parsimony consensus tree of exactly the same topology. Numbers behind branches refer to respective museum numbers **B** Parsimony network derived from the same alignment, with each node representing a unique haplotype separated by one substitutional step from its nearest neighbor. Rectangles are haplotypes of analyzed sequences, circles are haplotypes missing in our sample **C** Tentative taxonomic implication. Bar breaks indicate assumed species boundaries. Names refer to morphological determination or GenBank taxonomic identity.

The distinctiveness of *Diasporus citrinobapheus* is supported by the analysis of the 16S mitochondrial rRNA gene (Fig. 4). The four individuals we examined form a distinct cluster that appears separated from the other members for which 16S sequences are available. The mean genetic distance among the four specimens of *Diasporus citrinobapheus* is 0.3%. In our consensus tree (Fig. 4 A) it appears to be most closely related to the candidate species *Diasporus aff. diastema* from El Copé, from which it is separated by a mean genetic distance of 1.8%. In the haplotype network analysis (Fig. 4 B) both clades form unconnected subnetworks, indicating a differentiation at species level (Fig. 4 C). The mean genetic distance to the next closest relative *Diasporus quidditus* is 6.6% for *Diasporus citrinobapheus* and 7% for *Diasporus aff. diastema*.

#### Vocalization of holotype.

**Figure 5.**
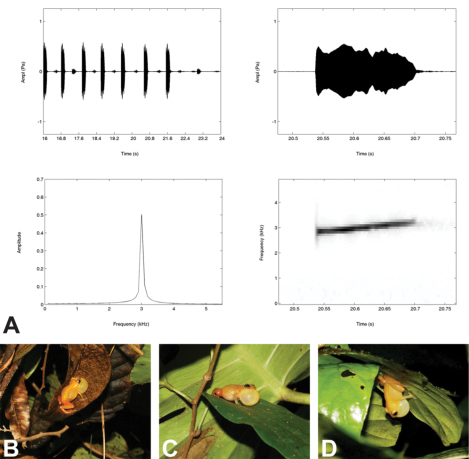
**A** Visualizations of an advertisement call (Hanning window function, FFT 512, 0.8 overlap) of *Diasporus citrinobapheus* (holotype, SMF 89814) recorded in Paredón, Comarca Ngöbe-Buglé, Panama, at 24.5°C air temperature and 95.3% relative humidity. Clockwise from top left: Oscillogram of a call group; Oscillogram of the penultimate call in the shown call group; Power spectrum showing the dominant frequency of the penultimate call in the shown call group; Spectrogram of the penultimate call in the shown call group **B–D** Different call positions of male *Diasporus citrinobapheus*: **B** Male holotype (SMF 89814) from Paredón calling on dead leaves in dense vegetation about 2 meters above ground level; **C** Male paratype (MHCH 2371) from Paredón on green leaf about 3 m above ground level **D** Male specimen (SMF 89817) from Willie Mazú referred to as *Diasporus citrinobapheus* calling from an elevated position on the underside of a leaf.

We recorded a 3 min, 43.5 seconds portion of the advertisement call of the holotype that yielded a total of 63 calls. An exemplary visualization of the call structure is given in Fig. 5 A. Relative humidity during recording was 95.3% at an air temperature of 24.5 °C. As in other members of the genus, the call consists of a single note, even though calls sound like a “whistle,” rather than the typical “tink” usually emitted by members of the genus *Diasporus* (Savage 2002; Chaves et al. 2009). The 63 recorded calls are organized in five call groups of 8–17 calls per group (12.8±3.2). A call group lasts 19.8–34.1 s (25.0±5.7). Intervals between call groups range from 15.7–33.2 s (21.6±8.0) and intervals between calls within a call group range from 0.57–5.77 s (1.93±1.2). Call group rate is 1.34 call groups per minute; call rate within a call group varies from 23.4–40.8 calls per minute (32.0±6.3). Call duration varies from 0.13–0.18 s (0.16±0.01). There is a rather weak frequency modulation of 190–470 Hz (370±65). The spectrum of frequencies within a call range from a mean minimum of 2890±44 Hz to a mean maximum of 3260±44 Hz. Fundamental and dominant frequencies are identical at about 2950 Hz. The dominant frequency, as the frequency with the greatest energy in the signal, is reached about 0.05 s after initiation of the call.

#### Vocalizations of paratypes and referred specimens.

In addition to the holotype, we recorded and analyzed the advertisement calls of two paratypes (SMF 89816, MHCH 2371) and one referred specimen (SMF 89817). Summing up, the advertisement call of *Diasporus citrinobapheus* sounds like a whistle, is organized in call groups, has a call duration of 0.14–0.16 s in average and a dominant frequency of 2860–3040 Hz (see all parameters in Table 2). While the paratypes vary only little in call parameters, SMF 89817 shows obvious differences regarding call duration, call interval, and call rate (see Discussion section for details).

**Table 2.** Variation in advertisement call parameters in four male specimens referred to as *Diasporus citrinobapheus* (mean±SD, min–max).

	**SMF 89814**	**SMF 89816**	**MHCH 2371**	**SMF 89817**
Temperature / RH during recording	24.5° C/95.3 %	24.3° C/93.5 %	24.6° C/93.6 %	21.8° C/100 %
Total recording time (min)	3.73	1.35	1.66	3.03
Number of call groups recorded	5	2	1	4
Number of calls recorded	63	26	11	68
Call group rate (call groups / min)	1.34	1.48	0.6	1.32
Call group duration (s)	25.0±5.7 19.8–34.1	23.0±9.5 16.3–29.7	19.0	20.6±8.5 12.5–28.5
Calls per group	12.8±3.2 8–17	11–15	11	16.6±5.7 10–22
Call group interval (s)	21.6±8.0 15.7–33.2	26.84	>78	25±16.7 10.9–43.5
Call rate over entire recording (calls/min)	16.9	19.3	6.63	22.4
Call rate within a call group (calls/min)	32±6.3 23.4–40.8	35.4±7.1 30.3–40.4	35	50±6.8 44.2–60
Call duration (s)	0.157±0.01 0.126–0.178	0.162±0.01 0.143–0.174	0.156±0.003 0.151 – 0.162	0.141±0.01 0.114–0.167
Call interval (s)	1.93±1.2 0.57–5.77	1.74±1.4 0.63–3.77	1.71±0.75 0.85 – 2.85	1.15±0.49 0.55–2.58
Dominant frequency (Hz)	2953±0	3010±75 2859–3140	2859±0	2965±32 2953–3046
Minimum frequency (Hz)	2889±44 2859–2953	2776±31 2765–2859	2671±0	2939±33 2895–2953
Maximum frequency (Hz)	3257±44 3140–3328	3184±61 3064–3234	3029±38 2953–3046	3290±74 3140–3421
Frequency modulation (Hz)	367 188–469	407 281–468	358 281–375	351 281–375

#### Geographical distribution and natural history notes.

**Figure 6.**
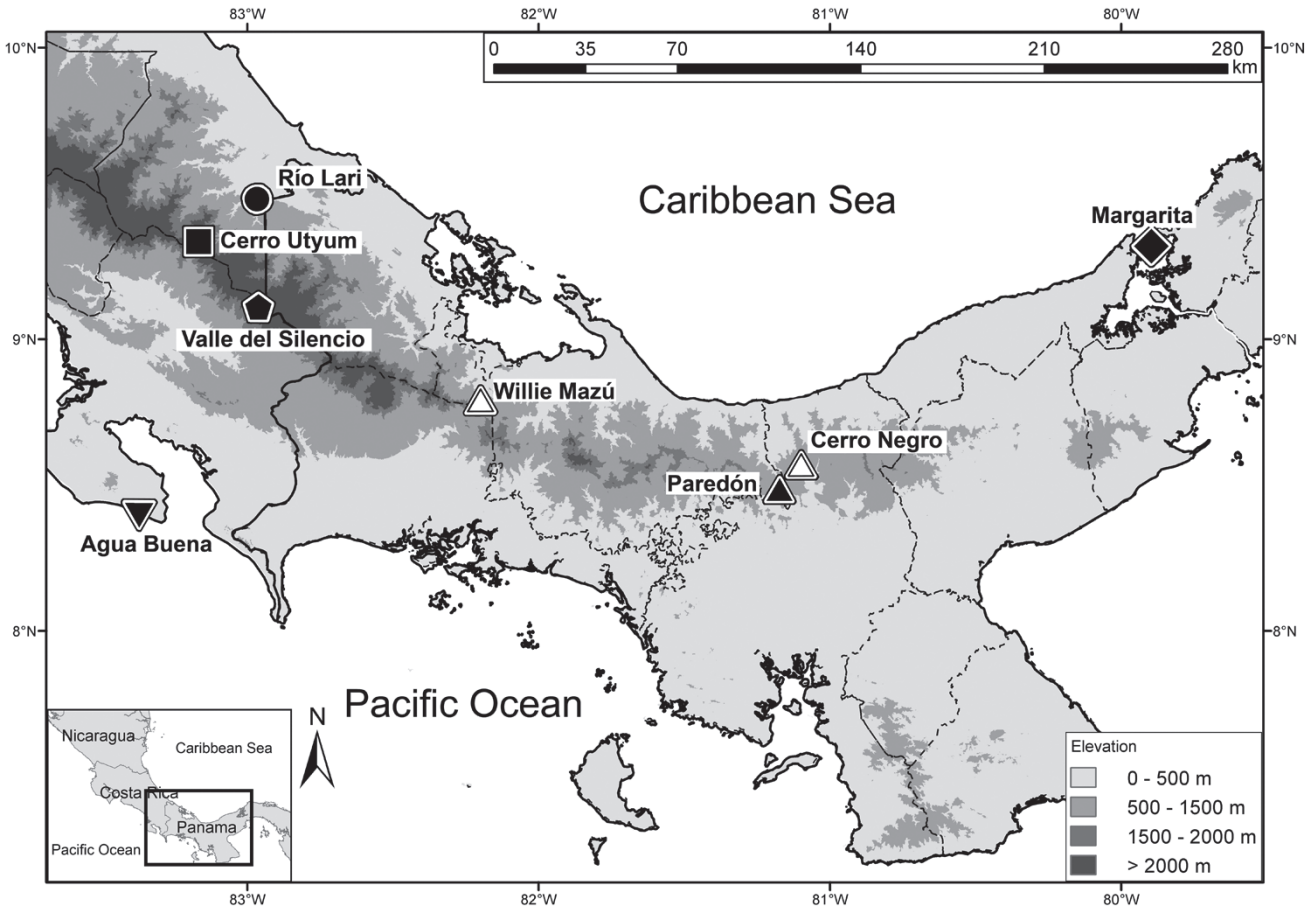
Distribution map of *Diasporus citrinobapheus* and type localities of other species in the genus in Panama and Costa Rica. **Solid triangle:** Paredón, Comarca Ngöbe-Buglé, type locality of *Diasporus citrinobapheus*. **Hollow triangles:** Additional collection sites of *Diasporus citrinobapheus*: Private Reserve Willie Mazú in the west, and Cerro Negro (Parque Nacional Santa Fé, Veraguas) in the east. **Inverted triangle:** Agua Buena, Puntarenas, Costa Rica, type locality of *Diasporus vocator*. **Pentagon:** Valle del Silencio, at the provincial boarder between Puntarenas and Limón, Costa Rica, type locality of *Diasporus ventrimaculatus*. **Square:** Cerro Utyum, Limón, Costa Rica, type locality of *Diasporus hylaeformis*. **Circle:** Río Lari, Limón, Costa Rica, type locality of *Diasporus tigrillo*. **Diamond:** Margarita, Colón, Panama, type locality of *Diasporus diastema*. Dashed lines represent provincial borders. Solid lines represent coast line and national border.

So far, *Diasporus citrinobapheus* has been found on the Caribbean slopes of the western Serranía de Tabasará and on both Pacific and Caribbean slopes of the eastern Serranía de Tabasará (Fig. 6) at intermediate elevations from 680 to 790 m.a.s.l. Males call from very dense vegetation and are difficult to spot. They are almost only detectable by following their characteristic vocalization. Vocal activity is highest just after dusk and finally stops when it becomes dark. Calling height ranges from near ground level up to three meters above ground. Calling position can be either on the upper side of a leaf or on its underside (Figs 4 B–D). The only female (SMF 89820) was found at daytime (15:00 h) inside an involute, young plantain leaf that apparently served as a daytime hiding place. The species does not seem to be limited to mature forest, but is also found in secondary growth and plantations. However, it appears to avoid open habitats like pasture land.

## Discussion

*Diasporus citrinobapheus* is easily distinguishable from all other known frogs of the genus in Lower Central America by its bright yellow to orange coloration. The only described species of the genus that somewhat resemble the new species in coloration are *Diasporus gularis* from Colombia and Ecuador and *Diasporus tigrillo* from the Caribbean slopes of the Costa Rican part of the Serranía de Talamanca. The latter species is known only from two specimens, both collected in 1964 at a single locality and there are no photographs of the species in life, tissue samples, or call recordings available to clarify the systematic relationships of this species. The different ground coloration in preservative between *Diasporus tigrillo* and *Diasporus citrinobapheus* is certainly due to different preservation techniques, because the fixation process in 10% formalin darkens the complete specimen. However, this does not influence the general color pattern, so we treat the dark brown spots on the dorsum of *Diasporus tigrillo* as a diagnostic feature to differentiate between *Diasporus tigrillo* and *Diasporus citrinobapheus*. Additional material is required, preferably from near the type locality of *Diasporus tigrillo* to conduct further studies. In contrast, *Diasporus gularis* is known from a number of specimens from Colombia and Ecuador. However, the presence and development of papillae at the apex of the pad on the underside of the disk cover, one of the main characters that has been used to distinguish this species from its congeners, is a controversial issue. Lynch (1976, page 12, Fig. 3 B) provided a drawing of the left hand of a specimen (LACM 73239) from Chocó, Colombia, that shows long papillae. In a later work, the same author presented drawings of finger disk pads of two *Diasporus gularis* (ICN 45168, 45171) from Valle del Cauca, Colombia, that show knobbed disk covers (Lynch 2001, Figs 2 A, B), but he also noted that there are specimens lacking this character. He further considered the presence or absence of this knob at the underside of the disk cover might be due to preparation technique. Lynch and Duellman (1997) noted that the holotype of *Diasporus gularis* from Ecuador does not have papillae at the tip of any digit, while they stated that specimens from southern Colombia and Ecuador have a rounded knob at the apex of the pad on the underside of the disk cover of toe II–IV. In Chocoan Colombia, specimens referred to as *Diasporus gularis* have larger papillae. Depending on their diagnosis, Lynch and Duellman (1997) argued that several species might currently be referred to *Diasporus gularis*, three in western Colombia and only one in Ecuador, where the type locality is. Based on our genetic analysis, *Diasporus citrinobapheus* is closely related to, and may even be conspecific with, the candidate species *Diasporus aff. diastema* from El Copé (Crawford et al. 2010a). Albeit the comparably small p-distance, the haplotype network analysis yields a separate network for each of the two clades, supporting the assumption of two distinct species. However, genetic evidence revealed only from mitochondrial markers alone is not strong enough to support either one or the other hypothesis (Vences et al. 2005). Further integrative taxonomic studies, including morphology, bioacoustics, and nuclear genes are needed to clarify this matter.

Besides various records of other amphibians and reptiles, we found no additional species of the genus *Diasporus* at the type locality. At Willie Mazú, a locality approximately 120 km NW of the type locality of *Diasporus citrinobapheus*, we collected a single specimen of *Diasporus* that we refer to *Diasporus vocator* based on size, coloration, disk shape, and male advertisement call. At Cerro Negro, *Diasporus citrinobapheus* occurs sympatrically with *Diasporus diastema*. Based on our current concept of its distribution, the possibility remains that also *Diasporus vocator* occurs at this locality, although the species has not been recorded from this site.

The eponymous, readily soluble yellow coloration of *Diasporus citrinobapheus* lead us to the assumption that this might serve a defensive function against predators. On this account, an alcohol extraction was analyzed for alkaloids, but no active substances were found. Probably, the yellow pigment is just highly soluble and therefore easily washed out. Nevertheless, one could speculate that it has a bitter or otherwise unpalatable taste that might deter certain predators.

Various studies have shown that the advertisement call represents a premating isolating mechanism in anurans (e.g., Duellman and Trueb 1986), which makes it a valuable tool in taxonomy. Having in mind that there are great morphological overlaps between members of the genus *Diasporus*, analyses of vocalizations might form the most powerful taxonomic approach to decipher its species diversity. Unfortunately, the calls of most species have never been formally described. Fouquette (1960) was the first to describe the call of *Diasporus diastema* from the Panama Canal area, about 10 km northwest of Panama City, not far from the type locality (Cope 1876; Dunn 1942). Later, Wilczynski and Brenowitz (1988) presented another call description based on calls recorded in the surroundings of Gamboa in Central Panama, about 24 km NNW of Panama City, and even closer to the type locality. Interestingly, the call descriptions of Fouquette (1960) and Wilczynski and Brenowitz (1988) are incongruent in terms of call duration, frequency range, and dominant frequency, rendering it possible that different species were recorded. Unfortunately, none of these papers cited any voucher specimens, so it is impossible to determine which species they actually recorded. The most recent contribution on vocalizations of *Diasporus diastema* is that of Ibáñez et al. (1999), also from the environs of the Panama Canal. They provided a rough sonogram, but did not give any numerical values. The dominant frequency in all three papers is roughly described as 3000–4000 Hz, thus considerably higher than in *Diasporus citrinobapheus*. Furthermore, all three papers (Fouquette 1960; Wilczynski and Brenowitz 1988; Ibáñez et al. 1999) present sonograms, which show an obvious frequency modulation expressed by a rapid rise of frequency over time with approximately 1000 Hz difference between beginning and end of the call. In contrast, the call of *Diasporus citrinobapheus* is characterized by only a moderate frequency rise over time, on average 350–400 Hz. Confusing are the data of call duration provided by Fouquette (1960) and Wilczynski and Brenowitz (1988), respectively. Fouquette (1960) reports on mean call duration of 0.2 s for *Diasporus diastema*. Yet, in the accompanying sonogram (Fig. 2 A in Fouquette 1960), the call seems to be only slightly longer than 0.1 s. Wilczynski and Brenowitz (1988) even mentioned a call duration of more than 0.3 s, but in the accompanying oscillogram (Fig. 1 B in Wilczynski and Brenowitz 1988), the call does not exceed 0.1 s on the time axis. Although difficult to assess precisely, the duration of the call pictured in the sonogram provided by Ibáñez et al. (1999) is clearly shorter than 0.2 s. Furthermore, Ibáñez et al. (1999) present a sonogram of *Diasporus vocator*, recorded also in the Canal Zone. According to their analysis, the call of *Diasporus vocator* has a frequency range between 6000 and 7000 Hz, is very short, and shows a strong frequency modulation, thus being very different from the calls of *Diasporus diastema* and *Diasporus citrinobapheus*. However, the type locality of *Diasporus vocator* is Agua Buena in the Peninsula de Osa, Costa Rica. Thus, it is advisable to record comparative call material from the type locality for future analyses. The most recent contribution on *Diasporus* vocalizations was made by Chaves et al. (2009) in the course of the original description of *Diasporus ventrimaculatus*. This species’ voice differs in all standard parameters from that of *Diasporus citrinobapheus*, as it has much shorter call durations of about 0.08 s, a low dominant frequency of about 2550 Hz, and a lower frequency range between 2140 and 2995 Hz. Furthermore, the dominant frequency is reached at the very beginning at the call. The same authors presented a preliminary analysis of calls emitted by specimens assigned to *Diasporus hylaeformis*. According to this analysis, call duration in *Diasporus hylaeformis* is on average 0.214 s, while it resembles *Diasporus ventrimaculatus* in spectral parameters. Regarding the vocalizations of *Diasporus tigrillo*, the least known species in the genus, only a field note citation appearing in the original description describes it as “similar to the dink dink of *Eleutherodactylus* [*Diasporus*] *diastema*” (Savage 1997).

Nevertheless, there is also an intraspecific variation among calls of specimens referred to *Diasporus citrinobapheus*. The call of the single male recorded at Willie Mazú (SMF 89817) differs from the calls of the members of the type series in temporal parameters, such as shorter call duration and call interval that result in a higher call rate. These differences are minor, but lead us to not include specimens from localities other than the type locality in the type series. However, various studies have shown that call parameter variation is linked to ambient temperature (e.g., Zweifel 1959; Schneider 1977; Gerhardt 1978). According to these studies, call duration and call interval are negatively correlated with temperature, which in turn leads to an increased call rate at higher temperatures. As SMF 89817 was recorded at lower temperatures than for the other three specimens, one would expect the opposite pattern. Nevertheless, these studies used data from many individuals, built scatter diagrams of parameters against temperature and fitted least-squares regression, and there are always outliers that do not follow the general trend. In our case, individual differences may be stronger than temperature-related ones, but this assumption needs further research to be reliably assessed.

Apart from morphology which apparently is not the best tool to identify species of *Diasporus*, neither DNA nor bioacoustics, both of paramount importance for contemporary anuran taxonomy, have been consistently analyzed among geographically and taxonomically wide-ranging samples. While the Panamanian and Costa Rican 16S barcodes compared in this study reveal the existence of more infrageneric lineages than names are available, the doubtless assignation of a given *Diasporus* “aff. *hylaeformis*” or “aff. *diastema*” is likely to be highly challenging if one is to rely on the existing treatments, which mostly provide only partial or even contradicting information. In conclusion, the complex and cryptic diversity within the genus *Diasporus* requires a thorough revision of as many “quality vouchers” (collected specimens associated with both well-preserved tissue samples and call recordings) from as many localities throughout the generic range as possible.

### Key to the species of *Diasporus* in Central America

**Table:** 

1a	Disk covers lanceolate or papillate	2
1b	Disk covers palmate or spadate	3
2a	Dorsum shagreened; fingers without thick lateral fringes; Toe V not partially fused with Toe IV; SVL of adult males 14.0–16.0 mm, of adult females 16.5–18.0 mm	*Diasporus vocator*
1b	Dorsum with scattered low warts; fingers with thick lateral fringes; Toe V partially fused with Toe IV; SVL of adult males 10.9–14.8 mm, of adult females 13.2–16.9 mm	*Diasporus quidditus*
3a	Fingers II and III with palmate disk covers and broadened, non-triangular disk pads; adults with vomerine teeth	*Diasporus diastema*
3b	Fingers II and III with spadate disk covers and triangular disk pads; adults with or without vomerine teeth	4
4a	Venter in most individuals with distinct black and white blotches; dorsum and dorsal surfaces of arms and legs in some individuals bright red in life	*Diasporus ventrimaculatus*
4b	Venter patternless or with a few small black dots; dorsum and dorsal surfaces of arms and legs brown, cream, or yellow in life	5
5a	Posterior surface of thigh pigmented (brownish, often suffused with red in life); overall dorsal coloration bright cream, grayish or reddish brown in life; adults without vomerine teeth	*Diasporus hylaeformis*
5b	Posterior surface of thigh unpigmented (yellow in life); overall dorsal coloration bright yellow to orange in life; adults with vomerine teeth	6
6a	Dorsum with scattered low pustules; ratio tympanum length / eye length 0.54–0.57; distal subarticular tubercle on Fingers and Toes I weakly bifid; dorsum yellow to orange with dark brown spots confined to pustules; SVL of adult males 16.0–17.5 mm	*Diasporus tigrillo*
6b	Dorsum smooth; ratio tympanum length / eye length 0.32–0.45; distal subarticular tubercle on Fingers and Toes I flat and rounded; dorsum uniformly bright yellow to orange, sometimes with irregularly distributed dark blotches; SVL of adult males 17.3–19.7 mm	*Diasporus citrinobapheus*

## Supplementary Material

XML Treatment for Diasporus citrinobapheus

## Appendix I

### Comparative material for morphological examination

*Diasporus diastema* — **Costa Rica:** Heredia: Puerto Viejo de Sarapiquí, entrance to La Selva, 30 m: SMF 81812; Rara Avis, 700 m: SMF 81811; **Honduras:** Gracias a Dios: Region La Mosquitia, Rio PlátanoBiosphere Reserve, Raudal Kiplatara, 162 m: SMF 85938; El Ocotilla, 410 m: SMF 85939; **Nicaragua:** Atlántico Norte: PN Saslaya, 920 m: SMF 82031-82035; Jinotega: Bosawas Biosphere Reserve: SMF 78561; National Park Saslaya, 188 m: SMF 78965; Matagalpa: Selva Negra, 1300 m: SMF 77231, 77235, 78184–78191; Río San Juan: Bartola, 30–70 m: SMF 80977–80979, SMF 79799–79800; Río Sarnoso, 25 m: SMF 79796–79798; Boca de San Carlos, 20 m: SMF 79794–5; ridge near Río Las Cruces, near Caño Negro, 415 m: SMF 83389; Cerro el Bolívar, near Río Machado, 280 m: SMF 83390; Lomas de Tambor, 210 m: SMF 83391; **Panama:** Bocas del Toro: Bosque Protector Palo Seco, 1148 m: SMF 84997; Archipelago Bocas del Toro, Isla Colón, 30 m: SMF 85068; Chiriquí: El Chorogo, 295 m: SMF 92008; Coclé: Cerro Gaitál, El Valle de Antón, 800 m: SMF 80781; Comarca Kuna Yala: Reserva Natural Nusagandi, 280 m: SMF 81961; Panamá: Canal Zone: SMF 29859, 29874.

*Diasporus aff. hylaeformis* — **Panama:** Bocas del Toro: Parque Internacional La Amistad, northern slope of Cerro Pando, 2400 m: SMF 89868, 89869, 89873, 89874, AH 263, AH 266. Chiriquí: Parque Internacional La Amistad, Jurutungo, southern slope of Cerro Pando, 2070–2330 m: SMF 89867, 89872, 89875, 89876, AH 126, 127; Cerro Punta, Las Nubes Ranger Station, 2070 m: SMF 89870, 89871.

*Diasporus vocator* — **Panama**: Bocas del Toro: Humedal de San San Pond Sak, 5 m: SMF 89865, AH 364; Comarca Kuna Yala: Reserva Natural Nusagandi, 350 m: SMF 81970–81976.

## Appendix II

**Table:** Corresponding information of sequenced *Diasporus* specimens.

Species	Collection number	Field number	GenBank accession no.	Country	Province	Latitude, Longitude
*Diasporus citrinobapheus*	SMF 89814	AH 449	JQ927333	Panama	Comarca Ngöbe-Buglé	8.485, -81.173
*Diasporus citrinobapheus*	SMF 89820	AH 211	JQ927334	Panama	Veraguas	8.569, -81.099
*Diasporus citrinobapheus*	MHCH 2370	AH 450	JQ927335	Panama	Comarca Ngöbe-Buglé	8.485, -81.173
*Diasporus citrinobapheus*	MHCH 2371	AH 452	JQ927336	Panama	Comarca Ngöbe-Buglé	8.485, -81.173
*Diasporus aff. hylaeformis*	SMF 89868	AH 267	JQ927337	Panama	Bocas del Toro	8.931, -82.714
*Diasporus aff. hylaeformis*	SMF 89869	AH 268	JQ927338	Panama	Bocas del Toro	8.931, -82.714
*Diasporus aff. hylaeformis*	SMF 89872	AH 124	JQ927339	Panama	Chiriquí	8.911, -82.713
*Diasporus aff. hylaeformis*	SMF 89875	AH 282	JQ927340	Panama	Chiriquí	8.912, -82.713
*Diasporus aff. diastema* ‘orange’	USNM 572442	KRL 0902	FJ784425	Panama	Coclé	8.667, -80.592
*Diasporus aff. diastema* ‘orange’	USNM 572443	KRL 1181	FJ784484	Panama	Coclé	8.667, -80.592
*Diasporus aff. diastema* ‘orange’	USNM 572454	KRL 0900	FJ784423	Panama	Coclé	8.667, -80.592
*Diasporus aff. diastema* ‘orange’	USNM 572455	KRL 0901	FJ784424	Panama	Coclé	8.667, -80.592
*Diasporus aff. diastema* ‘orange’	MVUP 1783	KRL 0694	FJ784338	Panama	Coclé	8.667, -80.592
*Diasporus aff. diastema* ‘orange’	MVUP 1830	KRL 0840	FJ784395	Panama	Coclé	8.667, -80.592
*Diasporus quidditus*	USNM 572444	KRL 0647	FJ784326	Panama	Coclé	8.667, -80.592
*Diasporus quidditus*	MVUP 1832	KRL 0856	FJ784405	Panama	Coclé	8.667, -80.592
*Diasporus vocator*	FMNH 257769	AJC 0127	JN991419	Costa Rica	Puntarenas	8.79, -82.96
*Diasporus aff. diastema*	USNM 572546	KRL 0782	FJ784369	Panama	Coclé	8.667, -80.592
*Diasporus aff. diastema*	MVUP 1826	KRL 0831	FJ784390	Panama	Coclé	8.667, -80.592
*Diasporus diastema*	MVZ 203844	1999	EU186682	Costa Rica	Cartago	9.75, -83.804
*Diasporus hylaeformis*	UCR 16264	AJC 0468	JN991418	Costa Rica	Alajuela	10.22, -84.54
*Pristimantis ridens*	UTA-A 57014	ENS 10722	JN991464	Honduras	Olancho	14.93, -86.14

## Appendix III

Audio samples of the advertisement calls of specimens of *Diasporus citrinobapheus*. (doi: 10.3897/zookeys.196.2774.app1) File format: MP3 Audio file (MP3).

Explanation note: MP3 audio samples of specimens of *Diasporus citrinobapheus* recorded at the type locality (Paredón) and Willie Mazú, Serranía de Tabasará, Panama.
